# Use of rosiglitazone before and after vascular injury in hypercholesterolemic rabbits: Assessment of neointimal formation

**DOI:** 10.1186/1477-9560-6-12

**Published:** 2008-08-27

**Authors:** Alexandre Alessi, Olímpio Ribeiro França Neto, Paulo Roberto Slud Brofman, Camila Prim, Lucia Noronha, Ruy Fernando Kuenzer Caetano Silva, Liz Andréa Villela Baroncini, Dalton Bertolim Précoma

**Affiliations:** 1 Center of Health and Biological Sciences, Pontifical Catholic University of Paraná, Brazil

## Abstract

**Objectives:**

To analyse the effects of rosiglitazone administered at different times on neointimal formation in hypercholesterolemic rabbits following vascular injury.

**Methods:**

Thirty-nine rabbits on a hypercholesterolemic diet were included. The animals underwent balloon catheter injury to the right iliac artery on day 14. They were divided into three groups as follows: control group, 13 rabbits without rosiglitazone; group I, 13 rabbits treated with rosiglitazone (3 mg/Kg body weight/day) for 28 days after the vascular injury; and group II, 13 rabbits treated with rosiglitazone (3 mg/Kg body weight/day) during all the experiment (42 days). Histological analysis was done by an experienced pathologist who was unaware of the rosiglitazone treatment. Histomorphometric parameters were performed by calculation of the luminal and intimal layer area, and intima/media layer area ratio (the area of the intimal layer divided by the area of the medial layer).

**Results:**

Intimal area was significantly lower in group II vs. CG (*p *= 0.024) and group I (*p *= 0.006). Luminal layer area was higher in group II vs. CG (*p *< 0.0001) and group I (*p *< 0.0001). Intima/media layer area ratio was equal between CG and group I. Intima/media layer ratio area was significantly lower in group II vs. control group (*p *< 0.021) and group I (*p *< 0.003). There was a significant reduction of 65% and 71% in intima/media layer area ratio in group II vs. control group and group I, respectively.

**Conclusion:**

Pretreatment with rosiglitazone in hypercholesterolemic rabbits submitted to vascular injury significantly reduces neointimal formation.

## Introduction

Peroxisome proliferator-activated receptor-γ (PPARγ) has been shown to be expressed in many of the cells that play a role in the response to vascular injury and to modulate the actions that are thought to initiate neointimal (NI) growth, including inflammation [1-4]. Neointimal formation is an important structural change in the vessel wall that leads to restenosis after angioplasty or stenting [5-8].

Thiazolidinediones consist of a family of synthetic compounds that acts as high-affinity ligands for PPARγ and were originally developed to facilitate glucose control in patients with type 2 diabetes. In addition, they have a direct impact on vascular cells and reduce circulating factors that are associated with atherosclerosis [9]. In a recent meta-analysis of randomized controlled trials there was evidence that thiazolidinedione therapy in patients undergoing coronary stent implantation may be associated with less in-stent restenosis and repeated revascularization [10-12]. Three different thiazolidinediones, rosiglitazone, pioglitazone, and troglitazone, have been shown to prevent balloon-injured rat carotid arteries [9]. Rosiglitazone can reduce the NI formation and macrophage content in a mouse injury model [1] and in hypercholesterolemic rabbits [2]. These effects were independent of glycemic control or changes in lipid concentrations [13]. In the present study we analyse the effects of rosiglitazone (RGZ) on neointimal formation administered at different times in hypercholesterolemic rabbits following vascular injury.

## Methods

### Animals

Thirty-nine white adult male rabbits (New Zealand), weighing 2.474 ± 348 Kg, were utilized for this experiment. Animals were handled in compliance with the Guiding Principles in the Care and Use of Animals. Protocol approval was obtained from the Pontifical Catholic University Animal Research Committee. During first 14 days the animals were fed a hypercholesterolemic diet (1% cholesterol-Sigma-Aldrich^®^). Subsequently, they were changed to a 0.5% cholesterol diet until sacrifice (42 days). The animals were divided into three groups as follows: control group (CG) 13 rabbits without RGZ; group I, 13 rabbits treated with RGZ from the fifteenth day (after the vascular injury) until sacrifice; and group II, 13 rabbits treated with RGZ during the entire experiment (42 days). Rosiglitazone was administered by oral gavage (3 mg/Kg body weight/day).

### Vascular injury

The rabbits underwent balloon catheter (20 × 3 mm/5 atm/5 min) injury of the right iliac artery on the fourteenth day of the experiment. Anesthesia was induced with ketamine (Vetanarcol^®^-König – 3,5 mg/Kg) and intramuscular xylazine (Coopazine^®^-Coopers – 5 mg/Kg). After the procedure the animals received intramuscular analgesics for 3 days (25 mg/day of flunixin – Banamine^® ^– Schering-Plough) and intramuscular antibiotics for 4 days (100 mg/day of oxitetraciclin – TormicinaP^®^-Toruga). The rabbits were sacrificed by a lethal barbiturate dose on day 42 and their aorta and iliac arteries were removed for immunohistochemical and histological analysis.

### Quantitative histopathology

Histological analysis was performed by an experienced pathologist (LN) unaware of the RGZ treatment. The analyses was done with a microscope attached to the Image Pro-plus^® ^4.5 Software (Media Cybernetics Inc. Silver Spring, MD. USA). Histomorphometric parameters were performed by calculation of the luminal and intimal layer area, and intima/media layer area ratio (the area of the intimal layer divided by the area of the medial layer) according to the method described by Phillips et al [1]. The quantification of total collagen was made by the Sirius red polarization method [14]. Atherosclerotic lesions were analysed and classified according to Virmani et al [15].

### Immunohistochemistry

Tissue preparation and immunohistological techniques were performed according to the manufacturer's instructions included in the kits (Dako Corporation, Carpinteria, Calif). Sections were stained for macrophage cells using primary monoclonal antibody RAM-11(Dako^®^, Carpinteria, CA), and for alpha-actin smooth muscle cells with primary polyclonal antibody HHF-35 (Dako^®^, Carpinteria, CA). For quantitative immunocytochemical comparisons of macrophage content or smooth muscle cell content in intimal area, sections were computed and scored in 2 categories based on less than or more than 50% of cells in the balloon injury area.

### Blood chemistry

Blood samples were obtained on first day of the experiment, immediately before balloon catheter injury, and immediately before sacrifice by cardiac puncture. Clinical laboratory assessment included fasting serum glucose, total cholesterol (TC), high-density lipoprotein cholesterol (HDL-C), and triglycerides (TGC). Measurements were done using an automated system (Abbott Architect ci8200; Abbott Laboratories, Abbott Park, Il).

### Statistical analysis

The calculation of sample size was done based on the study of Wang Zhao-hui, Luo Feng and Liu Xiao-mei [16]. The ratio between the intimal layer and the media layer was considered to be the main variable of interest. In order to detect a minimum difference of 0.15 between groups averages, with a significance level of 5% and power of the test of 80%, the minimum number of animals in each group of the study was defined as 12. Categorical variables were expressed as percentages and continuous variables were expressed as mean ± SD and medians. Data were compared using Anova one-way. The normality of the samples was tested by using Shapiro-Wilk tests. For non-normal samples, the Kruskal-Wallis and Mann-Whitney non parametric tests were used to compare the groups. Fisher's exact test was used for qualitative or categorical variables. Statistical significance was indicated by a value of *p *< 0.05. Analyses were performed using SPSS version 14.0 (SPSS, Inc., Chicago, Illinois).

## Results

### Metabolic and lipid profiles

The rabbit's weight did not differ between groups (data not shown). Baselineglucose, total cholesterol, HDL-cholesterol and triglycerides levels were equal in all groups before initiation of the diet. On day 14, two weeks after feeding, fasting glucose levels were higher in CG and group I. At the time of sacrifice glucose levels did not differ between groups. A graded elevation in TC and TGC levels was observed from the initial phase through the vascular lesion until sacrifice without significant differences between groups. A graded elevation in HDL-C was observed in all three groups. Higher levels of HDL-C were observed in group II versus CG and group I at the time of vascular injury and sacrifice (Table 1).

**Table 1:** Metabolic and lipid profiles (mean ± SD)

		CG	Group I	Group II	P value
Baseline	TC (mg/dl)	58.62 ± 25.08	50.77 ± 18.39	43.54 ± 14.82	NS
	HDL-C (mg/dl)	24.23 ± 5.31	23.1 ± 6.12	22.69 ± 6.26	NS *
	TGC (mg/dl)	79.62 ± 26.64	91.15 ± 32.71	79.85 ± 34.31	NS *
	Glucose (mg/dl)	121.38 ± 17.43	120.77 ± 19.07	117.92 ± 11.44	NS
Vascular injury	TC (mg/dl)	524.38 ± 258.94	431.77 ± 197.38	318.46 ± 212.86	NS
	HDL-C (mg/dl)	32.46 ± 19.66	28.77 ± 6.15	50.69 ± 21.91	NS *
	TGC (mg/dl)	72.77 ± 34.95	71.54 ± 46.54	86.92 ± 56.34	NS *
	Glucose (mg/dl)	250.23 ± 93.02	274.46 ± 58.45	166.62 ± 38.2	0.001
Sacrifice	TC (mg/dl)	852.46 ± 308.48	702.62 ± 261.53	593.54 ± 219,86	NS
	HDL-C (mg/dl)	42.62 ± 38.23	25.08 ± 13.93	69.08 ± 19.7	0,001 *
	TGC (mg/dl)	126.77 ± 85.66	398.08 ± 509.76	277.31 ± 248.14	NS *
	Glucose (mg/dl)	212.85 ± 73.71	210.92 ± 72.99	233.85 ± 89.65	NS

NS – non significant.

* Kruskal-Wallis

### Histomorphometry

**Figure 1:**
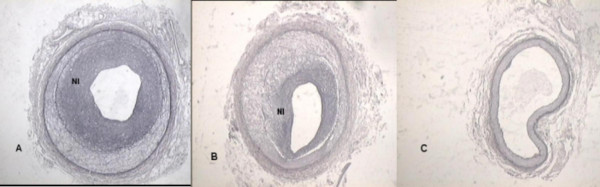
**Representative histological sections demonstrating neointimal formation – Orcein Staining.Panel A**: Control group. **Panel B**: Group I. **Panel C**: Group II. NI represents neointima.

**Figure 2:**
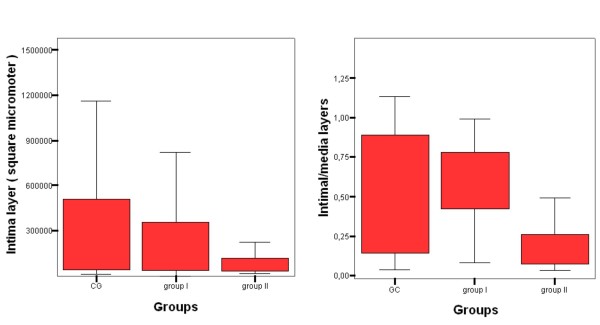
Quantification of intima/media layer area ratio; and total intimal layer area.

Intimal area was significantly lower in group II vs. CG (*p *= 0.024) and group I (*p *= 0.006). Luminal layer area was higher in group II vs. CG (*p *< 0.0001) and group I (*p *< 0.0001). There was a significant reduction of 65% and 71% in intima/media layer area ratio (IMR) in group II vs. CG (*p *= 0.021) and vs. group I (*p *= 0.003), respectively. Intima/media layer area ratio was equal between CG and group I. (Table 2). (Figures 1 and 2). According to the histological analysis proposed by Virmani et al, none of the criteria from1 trough 9 were found in group II, therefore the comparisons were only made between CG and group I. Neointimal growth, xanthomatous macrophages, proteoglican matrix, the presence and the thickness of fibrous cap, and the presence of calcification did not differ between CG and group I. There was no deposit of collagen into intimal or medial layers in group II, nor were there differences in the extent of collagen deposition between CG and group I. (Table 3).

**Table 2:** Quantitative histopathological analysis

Area	Group	Mean	DP	Minimum	Maximum	P
Intimal area	CG	320340.22	392880.74	14720.20	1512612.11	0.024
	GI	282659.14	346471.14	14500.40	1361362.80	0.006
	GII	83115.01	65440.66	16187.50	269226.60	
Luminal area	CG	458711.01	363013.82	1853.54	1773080.00	<0.0001
	GI	556450.31	330540.15	3274.59	1486461.00	<0.0001
	GII	861255.24	303153.71	222741.70	1586336.00	
IMR	CG	0.50	0.41	0.04	1.13	0.021
	GI	0.59	0.36	0.08	1.36	0.003
	GII	0.18	0.14	0.03	0.49	

IMR represents intima/media area ratio. Area was estimated in square micrometer.

**Table 3:** Qualitative histopathology between CG and Group I

	Presence	CG	Group I	*p *value
Intimal thickening (%)	No	30.76	15.38	> 0.05
	Yes	69.23	84.61	> 0.05
Isolated Xanthomamacrophages (%)	No	30.76	15.38	> 0.05
	Yes	69.23	84.61	> 0.05
Agregate Xanthomamacrophages (%)	No	38.46	15.38	> 0.05
	Yes	61.53	84.61	> 0.05
Lipid drops of proteoglican matrix (%)	No	38.46	15.38	> 0.05
	Yes	61.53	84.61	> 0.05
Lipid lakes of proteoglican matrix (%)	No	38.46	30.76	> 0.05
	Yes	61.53	69.23	> 0.05
Thin fibrous cap atheroma (%)	No	84.61	92.30	> 0.05
	Yes	15.38	7.69	> 0.05
Calcified nodule (%)	No	53.84	15.38	> 0.05
	Yes	46.15	84.61	> 0.05
Calcification (%)	No	92.30	76.92	> 0.05
	Yes	7.69	23.07	> 0.05
Collagen Type I (mean ± sd)		880.9 ± 436.5	264.5 ± 104.04	0.29
Collagen Type III (mean ± sd)		680.5 ± 267.54	312.24 ± 98.89	0.41

No represents less than 50% of cells in the balloon injury area. Yes represents more than 50% of cells in the balloon injury area.

### Immunohistochemistry

There was no significant difference in macrophage and smooth muscle cell content in the intimal layer between CG and group I (data not show). Group II did not present any intimal cell markers.

## Discussion

Prevention of restenosis after balloon coronary angioplasty or stent implantation with the use of local and systemic therapy is a challenging issue in interventional cardiology [4,17-20]. Osborne et al [21] showed that a short term model of hypercholesterolemia (two to four weeks) prevents extremely high cholesterol values and formation of advanced atherosclerotic plaques. Nevertheless, the arteries isolated from animals fed a cholesterol-enriched diet developed defects in endothelium-dependent relaxation in both large vessels as well as coronary resistance vessels [22]. These effects could be, in part, responsible for the restenosis after balloon angioplasty. Thiazolidinediones have immunomodulatory and antiproliferative effects, independent of their actions in metabolic control and are expressed in most cell types of the vascular wall as in atherosclerotic lesions, where they can affect atherogenic process [23-30]. To investigate the effects of a PPARγ ligand (rosiglitazone) on atherogenesis in an animal model, we used rabbits with six-fold increased cholesterol levels at the time of vascular injury and fourteen-fold increased levels at the time of euthanasia. This animal model was based on previous studies where rabbits develop hypercholesterolemia rapidly after excessive cholesterol feeding [2,3,22,24]. The metabolic effects of high cholesterol-containing diet on rabbits were extensively explained in our previous study [31]. Rosiglitazone was used at different times for each group. Group II not only did not present atherosclerotic lesions but also did not show any deposit of collagen or macrophage and smooth muscle cell markers in their intimal layer. The most significant findings were identified in the higher luminal area and the lower intimal area in which rabbits were treated with RGZ before vascular injury. Furthermore, in CG and group I intense reparative response occurred, with exuberant neointimal formation and reduction of luminal area. In addition, immunohistochemical analysis demonstrated a reduced macrophage and smooth muscle cell recruitment into the vascular arterial wall when RGZ was used two weeks before catheter balloon injury. Rosiglitazone did not exert anti-atherosclerotic activity when administered after vascular injury, however, a lesser density of macrophages in the media layer was observed in the animals of group I. We cannot rule out that these effects were due to chance, as our evaluation period was short. These findings suggest a possible protective effect of this drug against neointimal proliferation and remodeling responsible for restenosis after a balloon angioplasty. This is the first study to show the effects of a PPARγ ligand on vascular injury at different times and to document the benefits of pre-treatment with RGZ in hypercholesterolemic rabbits. Nevertheless, this drug has been the focus of extensive discussion in recent publications [32-37]. Nissen and Wolski [32] published a meta-analysis showing a significant increase in the risk of myocardial infarction and an increase in cardiovascular death of borderline significance in patients with diabetes receiving RGZ. Singh et al [33] also published a meta-analysis showing a significantly increased risk of myocardial infarction and heart failure among patients with impaired glucose tolerance or type 2 diabetes using rosiglitazone for at least 12 months, with no significantly increased risk of cardiovascular mortality. Lipscombe et al [34], in a nested case-control analysis of a retrospective cohort study, found that in diabetes patients with an age of 66 years or older, RGZ treatment was associated with an increased risk of congestive heart failure, acute myocardial infarction, and mortality when compared with other combination oral hypoglycemic agent treatments. The mechanism for the apparent increase in myocardial infarction and death from cardiovascular causes associated with RGZ remains uncertain. In the PERISCOPE randomized controlled trial [37], using coronary intravascular ultrasonography, the authors found a significantly lower rate of progression of coronary atherosclerosis in patients treated with pioglitazone when compared with glimiperide. However, it is not possible to extend the positive or negative benefit of one drug to another in the same class. In the next three years, we hope that the final results of the studies RECORD and BARI-2D [38], specifically evaluating cardiovascular effects of RGZ, will provide useful insights.

## Conclusion

The results of our study indicate that when rosiglitazone is administered in hypercholesterolemic rabbits before, but not after, undergoing vascular injury, there is significantly reduced neointimal formation.

## Competing interests

The authors declare that they have no competing interests.

## Authors' contributions

AA participated in the study design, ORFN participated in the study design, PRSB oriented in the surgical procedures, CP oriented in the management of the animals, LN made the histological examination, RFKCS oriented in the surgical procedures, LAVB wrote and oriented the manuscript, DBP participated in the study design. All authors read and approved the final manuscript.
